# Barriers and Facilitators of Tobacco Cessation Interventions at the Population and Healthcare System Levels: A Systematic Literature Review

**DOI:** 10.3390/ijerph22060825

**Published:** 2025-05-23

**Authors:** Sanchita Sultana, Joseph Inungu, Shayesteh Jahanfar

**Affiliations:** 1 Public Health Epidemiology, Washtenaw County Health Department, Ypsilanti, MI 48198, USA; 2 School of Health Sciences, Central Michigan University, Mount Pleasant, MI 48859, USA; inung1j@cmich.edu; 3 Public Health and Community Medicine, Tuft University School of Medicine, Boston, MA 02111, USA; shayesteh.jahanfar@tufts.edu

**Keywords:** tobacco cessation, health disparities, social determinants of health

## Abstract

Background: Tobacco use is responsible for eight million preventable deaths annually, making it a major modifiable risk factor for chronic conditions such as cardiovascular diseases, respiratory illnesses, and over 20 types of cancers. Objective: This study aimed to systematically review the barriers and facilitators of tobacco cessation interventions at both the population and healthcare system levels in the U.S. Understanding these determinants is critical for narrowing health disparities, optimizing resource allocation, and ultimately, enhancing tobacco cessation success rates across all demographic groups. Methods: A comprehensive literature search was conducted across the PubMed, Embase, and Web of Science databases, guided by the population, intervention, comparison, and outcome framework and quality assessment guided by PRISMA guidelines. Data extraction focused on study characteristics, intervention types, barriers, facilitators, and cessation outcomes at both the population and health system levels. The random effects forest plots were graphed to estimate pooled effect sizes for both medical and non-medical interventions. Results: A total of 35 studies met the inclusion criteria from an initial pool of 1555 identified records. Socioeconomic disadvantages, digital inequities, and low motivation constitute primary barriers at the individual level, while systemic factors such as healthcare access limitations, inadequate provider engagement, and lack of financial support further hinder cessation efforts. Financial incentives, culturally tailored interventions, and digital engagement strategies significantly improve tobacco cessation outcomes. Public health implications: as identified by the study, tailored interventions, the expansion of health coverage policies to include intervention, digital solutions, and healthcare resource workforce training will help improve tobacco cessation intervention outcomes.

## 1. Introduction

Globally, disparities in tobacco consumption exist among certain countries, with a few accounting for a significant proportion of consumers when compared to other developed countries. Tobacco is ingested by about 1.3 billion consumers globally; among these, 80% of consumers live in low- and middle-income countries [1]. Addictive tobacco consumption behavior is one of the causes of household poverty, as consumers prioritize its purchase over other essential needs like food or shelter. A total global health expenditure of about USD 1.4 trillion per year, along with productivity losses, are attributed to tobacco use, causing a significant economic burden, of which 40% is contributed by developing countries. In addition to the economic burden, there are significant preventable health risks related to tobacco consumption, leading to 8 million deaths annually. It is recognized as one of the preventable risk factors for chronic conditions such as cardiovascular and respiratory diseases, as well as 20 different types of cancers [1]. Cigarette smoking is one of the leading causes of preventable deaths in the U.S., killing about 480,000 of its consumers per year. In 2018, the U.S. economic burden due to tobacco consumption and its effect on health and productivity losses was estimated to be USD 600 billion [2].

Effective tobacco cessation interventions, including pharmacotherapy, behavioral counseling, and digital health solutions, are available; however, they remain underutilized and demonstrate less effectiveness in real-world settings compared to the results of randomized controlled trials (RCTs) [3]. Disparities in tobacco use are present among socioeconomically disadvantaged populations, racial minorities, and individuals facing adverse social determinants of health [4,5]. Disparities in utilization affect cessation treatment outcomes. Any treatment, while often tested in strict clinical environments, may be significantly influenced by real-world confounders when scaled for broad implementation. Understanding such contextual factors and whether they limit or support effectiveness can strengthen both causal analysis and the design of interventions aimed at narrowing tobacco-related health disparities. This review aims to establish the empirical foundation necessary to inform such evaluations and guide more equitable public health strategies. Therefore, it is important to identify these specific barriers [6,7]. At the population level, effective tobacco cessation interventions, i.e., behavioral counseling, alone or combine with pharmacotherapy, are available [8,9,10]. At the health system infrastructure level, various sources, such as face-to-face interactions, virtual messages, web-based platforms, or telephone interventions, exist to reach diverse populations and overcome challenges in implementation or population-level barriers. Several barriers are identified at both the individual and systemic levels, such as financial constraints, digital inequities, low motivation, limitations in healthcare access, and gaps in provider engagement, while identified facilitators, such as financial incentives, tailored interventions, social support, and improved healthcare infrastructure, play a crucial role in enhancing interventions’ effectiveness [11,12,13,14].

There are limited studies that comprehensively identify the barriers and facilitators of these extensive interventions and their implementation infrastructure [15,16,17]. However, these studies only focus on specific subpopulations or intervention modalities, which limits the generalizability of findings or the assessment of effectiveness in real-world settings. Furthermore, systematic evaluations of both medical (e.g., pharmacotherapy, nicotine replacement therapy) and non-medical (e.g., behavioral counseling, digital interventions) methods for overcoming cessation barriers are scarce. Understanding these factors is crucial for designing feasible and effective interventions in tackling tobacco use disparities. This review aims to synthesize the outcomes of medical and non-medical cessation interventions, while considering the population and health system level barriers and facilitators across diverse populations, settings, and implementation modalities in the U.S. While limited recent studies have explored the applications of evidence synthesis for constructing directed acyclic graphs (ESC-DAG) in other public health contexts [18], no work has leveraged systematic literature reviews to construct causally informed DAGs specific to tobacco cessation interventions. Additionally, as per a search across PubMed, Embase, Web of Science, and Google databases, there is no existing systematic literature review that assesses treatment effectiveness in the presence of barriers or facilitators across both the population and health system infrastructure level. Any treatment before implementation is tested in a strict clinical environment; however, its real-world implementation and effectiveness is affected by several confounders outside of a strict clinical environment. Identifying those confounders, i.e., barriers and facilitators, at both the receiving and implementation/facilitation levels can help strengthen future causal analysis. By systematically synthesizing barriers and facilitators across various intervention settings, this review aims to build the empirical foundation for developing ESC-DAGs that can enhance causal inference and statistical modeling in tobacco cessation research similar to the effect of limited existing research work in public health [18,19]. This is the first systematic review using a search across the previously mentioned databases to identify some of these confounding influences across the varied contextual factors of tobacco cessation treatment intervention receipt and implementation. Additionally, the review’s findings aim to guide public health policies and interventions regarding the real-world effectiveness of tobacco cessation interventions within the context of barriers and facilitators. For the same reason, this review does not focus on meta-regression analysis due to the presence of varied interventional modalities, along with the study design, study setting, and target population.

To clarify the scope of our review, we define “population level” as referring to barriers and facilitators experienced by individuals or groups receiving smoking cessation interventions, rather than to system-level implementation or provider-level factors. These include socio-demographic, behavioral, motivational, and contextual characteristics of target populations, especially among subgroups such as those with low socioeconomic status, racial and ethnic minorities, or individuals with mental health conditions. This distinction allows us to analyze how intervention effectiveness varies across end-user contexts, while differentiating between healthcare infrastructure or delivery issues.

Our review addresses a critical gap in the existing literature, where most studies assess barriers and facilitators within narrowly defined subpopulations or single intervention modalities. By synthesizing findings across both medical and non-medical interventions and examining these factors at both the individual and system levels, we aim to offer a broader and more actionable understanding of what influences tobacco cessation outcomes in real-world settings. This integrative approach allows for the identification of common barriers (e.g., digital inequity, mental health challenges) and facilitators (e.g., culturally tailored content, financial incentives) that recur across different implementation contexts.

## 2. Materials and Methods

### 2.1. PICO Framework

This systematic review followed the widely used population, intervention, comparison, and outcome (PICO) framework to guide study selection and synthesis [20]. The review focused on smokers residing in the U.S., evaluating tobacco cessation interventions that include behavioral therapies, pharmacotherapies, smoking cessation programs, and non-medical interventions. The primary outcome of interest was smoking cessation. PRISMA guidelines informed the reporting of study results.

### 2.2. Search Strategy and Database

A comprehensive literature search was conducted using the PubMed, Embase, and Web of Science databases on 1 December 2024. The gray literature source comprised connected papers [21], with manual bibliography searching. The citation management software used was Mendeley, version 1.19.5, released on 2018 [22], which helped identify duplicates. Similar Boolean terms and keywords were used across the identified database, and PICO framework guided the search strategy of the study. The search terms utilized across each databases are as mentioned in Appendix A Table A1, with search filters applied. Studies published between 2015 and 2025 were included in the screening for the final study sample.

### 2.3. Screening Questions

Two reviewers, S.S. and J.I., independently assessed and screened the studies, and any disagreements were resolved through discussion. In case of unresolved disagreement, a third reviewer, J.S., was consulted to reach a conclusive resolution. We used the following screening questions to determine whether to include or exclude studies. If a study met the following criteria during title, abstract, and body screening, it was included in the final sample.

1. Is it about tobacco/smoking cessation interventions AND
2. Is it conducted in the United States AND
3. Is the study outcome tobacco cessation AND
4. Are health/healthcare barriers and facilitators explored?

Barriers and facilitators to smoking cessation intervention implementation or outcomes were identified through inductive qualitative analysis of the Discussion and Results sections of each included study. Statements made by authors to explain their findings, i.e., challenges regarding recruitment, contextual factors, cultural relevance, acceptability, and resource availability, were extracted and coded. These were then categorized as barriers or facilitators, based on their influence on the intervention’s feasibility, effectiveness, or uptake.

### 2.4. Data Extraction, Analysis, and Quality Assessment

A standardized data extraction form was used to collect study details, including the author, study design, intervention type, population characteristics (i.e., smokers with mental illness, other conditions, or pregnancy), sample size, identified barriers, facilitators, and cessation outcomes. This review aimed to categorize the barriers and facilitators of both medical and non-medical tobacco cessation interventions at the population and healthcare system levels. A forest plot was created to pool the effect estimates of cessation interventions. Cochran’s Q test and the I-squared statistic were used to assess the heterogeneity of the included studies. A random-effects model was employed to account for variability across studies.

The quality assessment of the included studies was performed using a quality checklist for both RCT and non-RCT health intervention studies [22]. The quality assessment criteria included statistical conclusion validity, construct validity, i.e., power, reporting, and external validity, and internal validity, i.e., confounding and bias.

Barriers and facilitators were identified using inductive qualitative analysis. We extracted explanatory text from the Discussion, Limitations, and Conclusion sections of each study, in which authors interpreted significant or non-significant outcomes in light of contextual or structural factors. These factors were then coded and categorized based on whether they appeared to enhance (facilitators) or inhibit (barriers) the intervention’s impact or reach. This process enabled the synthesis of findings, even when the original study’s primary objective was not to evaluate barriers and facilitators directly. We extracted information on reported barriers and facilitators toward intervention implementation for the sub-population in the required studies. Barriers and facilitators were identified from both quantitative findings and qualitative content, including authors’ interpretations, contextual descriptions, and discussion of implementation issues, even when these factors were not directly assessed or statistically tested. This approach allowed us to capture relevant insights from all included studies, including those that did not demonstrate statistically significant differences in cessation outcomes.

A qualitative content analysis approach was used to synthesize these factors, examining how they were described and emphasized in each study. Common barriers and facilitators were identified based on both their recurrence across studies and the degree of emphasis placed on them by study authors, either regarding the results or the interpretation of the findings.

## 3. Results

**Figure 1:**
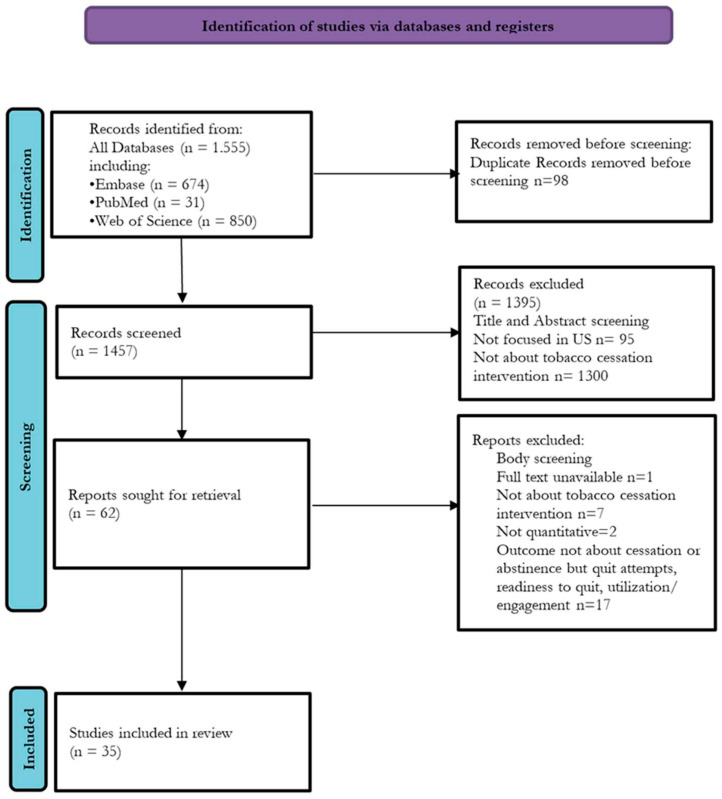
PRISMA flowchart.

A total of 35 studies met the inclusion criteria from an initial pool of 1555 identified records (Figure 1). Among these, 19 studies examined barriers and facilitators at the population level, with 10 assessing medical interventions and 9 evaluating non-medical interventions (Table 1A,B). Additionally, 16 studies focused on barriers and facilitators at the healthcare system level, with 6 investigating medical interventions and 10 exploring non-medical interventions (Table 2A,B).

### 3.1. Population Level Barriers and Facilitators in Tobacco Cessation Interventions

#### 3.1.1. Medical Interventions

The identified studies employed various medical interventions, including combined pharmacotherapy and behavioral counseling, financial incentives for pharmacotherapy, digital cessation interventions along with pharmacotherapy, or a combination of these with usual care (Table 1A). Key barriers included socioeconomic disadvantages such as homelessness, financial constraints, digital inequities, and mental or emotional issues like mental illness and low motivation. Facilitators that contributed to improved cessation outcomes encompassed financial incentives, peer motivation and social support, the incorporation of multilingual components in interventions for culturally diverse populations, patient-tailored therapies, nicotine replacement therapy (NRT), any combination of other therapies, integrated treatment services for tobacco cessation, or the implementation of digital intervention methods (Table A2).

The included studies assessed the effectiveness of various smoking cessation interventions among socioeconomically disadvantaged and high-risk populations. Effect sizes were reported as odds ratios (OR) or relative risks (RR), with corresponding 95% confidence intervals (CIs), reflecting the magnitude and statistical significance of each intervention’s impact. Interventions incorporating financial incentives demonstrated the strongest effects on smoking cessation. Baggett et al. reported an OR of 8.08 (95% CI: 3.35–19.5) for a program combining financial incentives, nicotine replacement therapy (NRT), and counseling among homeless smokers, indicating a highly significant effect. Similarly, Higgins et al. found that financial incentives combined with NRT increased cessation rates among socioeconomically disadvantaged mothers, with an OR of 5.88 (95% CI: 1.87–18.48). Kendzor et al. also reported a significant effect of financial incentives for low-income adults, with an OR of 3.18 (95% CI: 1.70–5.95). Community-based and behavioral interventions also demonstrated positive effects. Brooks et al. evaluated a community advocate-led cessation program for public housing residents, reporting an OR of 2.98 (95% CI: 1.56–5.68). Hooper et al. examined a culturally adapted video–text intervention combined with NRT among African American smokers, yielding an OR of 3.02 (95% CI: 0.53–7.37), although the wide CI suggests uncertainty in the estimate. Halpern et al. found that financial incentives combined with pharmacotherapy resulted in an OR of 2.27 (95% CI: 1.24–4.17), demonstrating statistical significance. Interventions targeting language barriers and mental health conditions showed varied effectiveness. Chen et al. assessed Asian-language behavioral counseling and NRT among Asian immigrants, reporting an OR of 0.40 (95% CI: 0.36–0.45), indicating reduced cessation rates compared to those for the controls. Meernik et al. examined a community-based cessation program for individuals with behavioral health conditions and found a modest effect (OR = 1.15, 95% CI: 1.08–1.21). Hickman et al. assessed a transtheoretical model-based intervention tailored for uninsured smokers with mental illness, reporting an OR of 1.80 (95% CI: 0.74–4.38), although the wide CI suggests limited precision. Finally, a mobile phone-delivered cessation intervention combined with NRT was evaluated by Vidrine et al. among socioeconomically disadvantaged smokers, yielding a relative risk of 2.11 (95% CI: 1.00–4.48), suggesting potential effectiveness, although with borderline statistical significance.

#### 3.1.2. Non-Medical Interventions

Non-medical interventions at the population level included acceptance and commitment-based behavioral therapy implemented through a smartphone app, patient-tailored interactive and non-interactive text and web interventions, and cognitive behavioral counseling on its own (Table 1B). Common barriers included digital inequities, low motivation, digital literacy disparities, stress, stigma, household smoking exposure, being a racial minority, being a daily heavy smoker, and having less than a high school education. Effective facilitators consisted of behavioral support, self-paced engagement, personalized messaging, motivational support, cognitive behavioral therapy interventions, stricter indoor smoking restrictions, and the implementation of virtual interventions.

The efficacy of various smoking cessation interventions was evaluated across multiple randomized controlled trials (RCTs) and one cohort study, with effect sizes reported as the relative risk (RR) or odds ratio (OR), along with 95% confidence intervals (CIs). Bricker et al. (RR = 1.72, 95% CI: 1.45–2.05) demonstrated a significantly increased cessation rate among general adult smokers using an ACT-based smartphone application compared to the use of a standard app, indicating a robust effect. Similarly, Lee et al. (OR = 2.53, 95% CI: 1.21–5.28) found cognitive behavioral counseling (CBC) to be an effective intervention for pregnant and postpartum women, with a more than twofold increase in cessation likelihood. Mays et al. reported a significant effect of tailored mobile messaging for waterpipe smokers (OR = 1.9, 95% CI: 1.1–3.3), suggesting that personalized SMS interventions could be beneficial. Graham et al. (OR = 1.39, 95% CI: 1.15–1.68) and Villanti et al. (RR = 1.09, 95% CI: 1.04–1.15) reported significant, but comparatively modest, effects for mobile-based interventions targeting young adult e-cigarette smokers and socioeconomically disadvantaged young adults, respectively. Collins et al. (OR = 11.0, 95% CI: 6.3–19.2) reported the highest effect size, with behavioral counseling in mothers of young children associated with significantly increased smoking cessation, likely driven by household smoking restrictions. Conversely, Heffner et al. (OR = 0.91, 95% CI: 0.65–1.28) found no significant difference in cessation rates between sexual minority and nonminority smokers in web- and text-based interventions, suggesting that additional barriers such as minority stress and stigma may attenuate intervention effectiveness. Christiansen et al. also reported no significant difference in smoking cessation outcomes among low-socioeconomic status (SES) smokers receiving brief interventions in community settings. Similarly, Kamke et al.’s cohort study of text-based cessation interventions for pregnant women (OR = 1.01, 95% CI: 0.57–1.78) showed no significant impact, particularly when stratified by race and education level.

### 3.2. Healthcare System Level Barriers and Facilitators in Tobacco Cessation Interventions

#### 3.2.1. Medical Interventions

Several studies identified medical interventions facilitated by healthcare system infrastructure, such as NRT, a combination of pharmacotherapy and proactive outreach, tobacco clinics in public housing, quitline services, and services involving community health workers for implementation (Table 2A). Healthcare system-level barriers included treatment access issues, rurality, cost of care, lack of provider engagement, and living in areas with limited health resources. Facilitators identified in the studies included free treatment availability and access, proactive encouragement, implementation of comprehensive treatments (a combination of pharmacotherapy and behavioral therapy), community-based clinic support, and social support provided by healthcare workers.

Among the randomized controlled trials (RCTs) reviewed, Dahne’s study assessing nicotine replacement therapy (NRT) sampling among general smokers reported an OR of 1.59 (95% CI: 0.97–2.59). Although suggestive of a positive effect, the confidence interval was 1.0, meaning the result is not statistically significant. In contrast, Fu et al. examined proactive outreach tobacco treatment among low-income smokers covered by Medicaid or MinnesotaCare, reporting an OR of 1.47 (95% CI: 1.12–1.93). The significant confidence interval suggests that proactive treatment, including free access to NRT and behavioral counseling, increases cessation rates in this population. Lee et al. investigated the impact of cessation advice receipt among U.S. adult smokers, finding an OR of 0.99 (95% CI: 0.95–1.00), indicating no meaningful association. Similarly, Kerkvliet et al. reported an OR of 0.78 (95% CI: 0.68–0.89) when examining quitline services combined with cessation treatment among smokers with and without mental health conditions. The significant negative effect suggests that additional psychological barriers may hinder the effectiveness of quitline interventions. Wewers et al. evaluated cessation treatment that involved community health workers and NRT among Appalachian smokers, finding an OR of 1.04 (95% CI: 1.01–1.06). While statistically significant, the effect size is modest, indicating only a slight improvement in cessation outcomes.

#### 3.2.2. Non-Medical Interventions

System-level non-medical interventions included smartphone-based incentives, policy changes, i.e., Affordable Care Act (ACA) Medicaid expansion for tobacco treatment cessation coverage, and social media-based cessation intervention implementation (Table 2B). Common barriers included limited access to healthcare due to insurance coverage, observed racial discrimination within the health system, low awareness of pharmacotherapy, cultural obstacles, geographic isolation, digital access challenges, and various individual socioeconomic, mental, and emotional disadvantage factors. Facilitators included smartphone-based incentivized interventions; Medicaid expansion coverage for treatment; online peer and counselor support; automated prompts; specialized quitlines; live counseling engagement; greater acceptance of physical sensations, emotions, and thoughts; digital interventions; and coping response training.

Kurti et al. reported a substantial effect of a smartphone-based incentivized intervention for pregnant smokers, yielding an odds ratio (OR) of 9.33 (95% CI: 1.87–46.68), indicating a strong association between the intervention and smoking cessation. Similarly, Chen Lyu et al. found that social media-based cessation interventions for young adult smokers were associated with increased cessation likelihood (OR = 2.6, 95% CI: 1.8–4.2), despite identified digital access barriers. Bailey et al. investigated the impact of Medicaid expansion on smoking cessation among Medicaid-insured smokers and observed a modest but significant effect (OR = 1.35, 95% CI: 1.28–1.43), suggesting that healthcare access plays a critical role in cessation outcomes. Hooper, M.W. et al. examined cessation treatment incorporating cognitive-behavioral therapy (CBT) and generalized health education (GHE) among individuals facing racial barriers, with results showing an OR of 0.97 (95% CI: 0.94–0.99), reflecting a minimal but statistically significant effect. In studies evaluating digital interventions, Santiago-Torres et al. assessed the iCanQuit app for American Indian and Alaska Native smokers, yielding an OR of 1.97 (95% CI: 0.92–4.25), suggesting a potential but inconclusive effect. Similarly, an ACT-based smartphone cessation intervention for rural smokers resulted in an OR of 1.47 (95% CI: 0.96–2.27), indicating moderate efficacy. McCarthy et al. explored the effectiveness of an electronic health record (EHR)-enabled smoking treatment program among primary care patients, reporting a wide confidence interval (95% CI: 2.2–10.5), although the specific OR was not provided. Kreuter et al. found that specialized quitline services with navigation support for low-income smokers had a significant but negative effect on cessation (OR = 0.70, 95% CI: 0.50–0.95), implying that barriers such as low awareness of nicotine replacement therapy (NRT) and financial constraints may hinder cessation success. Thrul’s study on Facebook-based live counseling for young adult smokers demonstrated a small but positive impact (OR = 1.10, 95% CI: 1.02–1.20), emphasizing the role of social support in cessation. Webb Hooper et al. investigated CBT combined with an eight-week nicotine patch regimen in treatment-seeking smokers and reported an OR of 0.93 (95% CI: 0.89–0.98), indicating a small but statistically significant reduction in smoking likelihood associated with reductions in perceived distress and depressive symptoms.

## 4. Discussion

The study identifies key factors influencing the effectiveness of multifaceted tobacco cessation interventions at both the population and healthcare system levels. Common barriers include limited access to healthcare, digital literacy gaps, and psychological challenges, which hinder engagement with cessation programs. In contrast, facilitators that enhance tobacco cessation outcomes include tailored motivational support, behavioral therapy, expanded treatment coverage, improved healthcare access, and social support.

The findings suggest that integrating tailored interventions with expanded access to treatment can optimize cessation outcomes by addressing both individual and systemic barriers. Socioeconomic disadvantage, stress, low motivation, and mental illness significantly impact cessation success at the individual level. At the healthcare system level, structural and process-related components, i.e., healthcare infrastructure, provider engagement, and policy-driven interventions, play a critical role in ensuring equitable access to cessation treatments. A comprehensive approach that simultaneously tackles these barriers at multiple levels is essential for reducing disparities in treatment outcomes.

While financial incentives were identified as effective facilitators of smoking cessation in several studies, we acknowledge that their impact was often limited to short-term follow-ups. For example, studies such as those by Baggett and colleagues demonstrated significant cessation effects at early timepoints, but these effects tended to diminish over time [23,28]. Therefore, financial incentives should be interpreted as short-term facilitators that enhance initial cessation rates, particularly among highly disadvantaged populations, rather than as sustained solutions. Long-term effectiveness may require continued support or complementary behavioral interventions.

Multilingual services, materials, or personnel were noted as facilitators in several studies. These components were intentionally designed to address language barriers and improve cultural and linguistic alignment, thereby facilitating access to cessation services among racial and ethnic minoritized groups. We categorized such components as facilitators based on their intended function to promote engagement and inclusivity, rather than based solely on cessation outcomes. For instance, although a study [24] reported lower cessation rates among users of multilingual quitlines compared to those for English-speaking users, this comparison involved different cohorts across distinct time periods (2004–2008 vs. 2012–2019), with potential confounding factors. Despite the cessation outcome, the multilingual service model was introduced specifically to address known language and access disparities, aligning with our definition of facilitators in this review.

This study acknowledges certain limitations. The high heterogeneity observed across studies suggests variability in intervention effectiveness, likely influenced by differences in study design, population characteristics, and implementation contexts. While the random-effects model in the pooled analysis accounts for some degree of heterogeneity, future research should conduct stratified analyses to identify the most impactful intervention components for specific subpopulations. Additionally, publication bias and the reliance on self-reported cessation outcomes in some studies may limit the generalizability of the findings. Further studies employing objective cessation measures and longitudinal analyses are needed to strengthen the evidence base for effective tobacco cessation strategies. Our inclusion of the work of [12] reflects the broader scope of this review to capture structural and policy-level changes that function as population-level interventions. While the study is observational and subject to baseline differences between comparison groups, its difference-in-differences design supports a tentative association between Medicaid expansion and increased cessation, particularly among Hispanic populations. Nonetheless, we acknowledge the limitations in attributing causality due to potential confounding and lack of randomization.

It is essential to note that while our synthesis primarily includes studies focused on specific subpopulations, such as individuals of low socioeconomic status (SES), racial/ethnic minorities, or pregnant women, our objective was not to achieve national representativeness, but rather to integrate insights from diverse real-world implementation contexts. These contexts often overlap with priority populations disproportionately burdened by tobacco use, and the repetition of certain themes across multiple studies provides confidence in the generalizability of key barriers and facilitators. We acknowledge that this approach may not fully capture the experiences of higher SES or low-risk populations, as they are underrepresented in the literature. Nonetheless, by aggregating findings from a wide range of studies, our review offers a comprehensive overview of the most persistent challenges and enablers across healthcare and behavioral domains.

This review contributes to the field by addressing limitations in previous work that either aggregated diverse populations or focused exclusively on intervention efficacy. By synthesizing implementation barriers and facilitators across studies involving racially/ethnically minoritized and SGM populations, our review uniquely centers equity-related contextual and structural considerations. Unlike prior reviews, this study also includes both clinical and policy-level interventions, highlighting how systems-level changes like Medicaid expansion which may influence cessation in underrepresented groups. The originality of this review lies in its focus on implementation contexts, its explicit attention to subgroup-specific dynamics, and the integration of findings across heterogeneous intervention types, offering practical insights for equity-focused program design and future research.

## 5. Recommendations for Public Health and Healthcare Systems

This review established the significance of a multi-tiered approach to tobacco cessation interventions that considers both individual and systemic determinants. Improving health care access, workforce engagement, and digital solutions can help achieve the effectiveness of cessation in a real-world setting. Prioritizing digital health, telemedicine, mobile health, text messaging or relevant virtual modes of implementation can help improve access, particularly for populations facing geographic and healthcare access barriers. Incorporating culturally and linguistically tailored interventions can help improve treatment reach and effectiveness among culturally diverse and some hard-to-reach target populations. Finally, improving healthcare workforce training in inculcating cessation interventions during routine practices can help strengthen cessation treatment utilization, engagement, and outcomes.

## Figures and Tables

**Table 1:** (A) Population level medical intervention study characteristics. (B) Population level non-medical intervention study characteristics.

(A)								
Author	Study Design	Intervention	Study Population	Sample Size	Identified Barriers	Identified Facilitators	Cessation Outcomes (Effect, 95% CI)	Quality Rating
Baggett et al. [23]	RCT	Financial Incentives with nicotine patch and counseling	Homeless smokers	50	Homelessness	Financial incentive	OR = 8.08 (95% CI: 3.35–19.5)	High
Brooks et al. [8]	RCT	Community advocate-led cessation	Public housing residents	250	Socioeconomically disadvantaged	Peer motivation and social support	OR = 2.98 (95% CI: 1.56–5.68)	High
Chen et al. [24]	Cohort	Asian language behavioral counseling+ NRT	Asian immigrants	14,073	Language barriers	Multilingual quitline services+ NRT/e-cigs	OR = 0.40 (95% CI: 0.36–0.45)	Moderate
Hickman et al. [10]	RCT	Transtheoretical model (TTM)-tailored, computer assisted intervention	Uninsured low SES smokers with mental illness	100	Financial constraints, mental illness	Tailored therapy	OR = 1.80 (95% CI: 0.74–4.38)	High
Higgins et al. [25]	RCT	Best practice + financial incentives + NRT	Mothers of children < 12 yrs old	198	Socioeconomically disadvantaged	NRT+ Incentive	OR = 5.88 (95% CI: 1.87–18.48)	High
Hooper, M.W. et al. [26]	RCT	Culturally adapted video–text tobacco cessation intervention+ NRT	Economically disadvantaged and African Americans	119	Digital inequities	High motivation, NRT	OR = 3.02 (95% CI: 0.53–7.37)	High
Halpern [27]	RCT	Financial incentive programs+ usual care + pharmacotherapy	General smokers	2538	Not identified	Incentives for abstinence	(OR = 2.27, 95% CI: 1.24–4.17)	High
Kendzor et al. [28]	RCT	Financial incentives for low-income adults+ usual care and medication	Socioeconomically disadvantaged smokers	320	Socioeconomic barriers	Financial rewards	OR = 3.18 (95% CI: 1.70–5.95)	High
Meernik et al. [29]	Cohort	Community-based cessation program + NRT/other medication	Smokers with behavioral health conditions	974	Mental health barriers	Integrated treatment services	OR = 1.15 (95% CI: 1.08–1.21)	Low
Vidrine et al. [30]	RCT	Mobile phone-delivered Cessation+ NRT	Socioeconomically disadvantaged smokers	624	Low motivation, socioeconomic barriers	NRT+ Text+ Call	RR = 2.11 (95% CI 1.00–4.48)	High
**(B)**								
**Author**	**Study Design**	**Intervention**	**Study Population**	**Sample Size**	**Identified Barriers**	**Identified Facilitators**	**Cessation Outcomes (Effect, 95% CI)**	**Quality Rating**
Bricker [31]	RCT	ACT-based smartphone app vs. standard app	General adult smokers	2088	Digital access, motivation variability	Self-paced engagement, behavioral support	(RR = 1.72, 95% CI: 1.45–2.05)	High
Villanti [32]	RCT	Tailored text message and web intervention	Socioeconomically disadvantaged young adults	437	Digital literacy barriers	Personalized messaging, motivational support	(RR = 1.09, 95% CI: 1.04–1.15)	High
Heffner [33]	RCT	Web+text-based cessation interventions	Sexual minority vs. nonminority smokers	2637	Minority stress, stigma	Not identified	(OR = 0.91, 95% CI: 0.65–1.28)	High
Mays et al. [34]	RCT	Tailored mobile messaging for waterpipe cessation	Young adult waterpipe smokers	349	Low motivation	Personalized SMS support	OR = 1.9 (95% CI: 1.1–3.3)	High
Lee, M. et al. [35]	RCT	Cognitive behavioral counseling Cessation	Pregnant and postpartum women	277	Psychological stress	CBC intervention	OR = 2.53 (95% CI: 1.21–5.28)	High
Christiansen et al. [36]	RCT	brief intervention in Wisconsin Salvation Army sites	Low SES smokers	522	Low motivation to quit	Community support	No significant difference between control and intervention	High
Collins et al. [37]	RCT	Behavioral counseling	Mother of young children < 4 years old	300	Household smoking exposure	Stricter indoor smoking restrictions	OR = 11.0 (95% CI: 6.3–19.2)	High
Graham et al. [38]	RCT	Vaping cessation interactive text messaging	Young adult e-cig smokers age 18–24	2588	Low motivation	Mobile-based intervention	OR = 1.39 (95% CI: 1.15–1.68)	High
Kamke et al. [39]	Cohort	Text-based smoking cessation for pregnant women	Pregnant smokers	1288	Race, less than high school education, daily smokers	No racial significant difference	OR = 1.01 (95% CI: 0.57–1.78)	Moderate

**Table 2:** (A) Health system level medical intervention study characteristics. (B) Health system level non-medical intervention study characteristics.

(A)								
Author	Study Design	Intervention	Study Population	Sample Size	Identified Barriers	Identified Facilitators	Cessation Outcomes Effect, 95% CI	Quality Rating
Dahne [40]	RCT	Nicotine replacement therapy sampling	General smokers	1245	Access barriers, race, income, rurality, education	Free provision, proactive encouragement	OR = 1.59 (95% CI: 0.97–2.59)	High
Fu et al. [41]	RCT	Proactive outreach tobacco treatment	Low-income smokers covered by Medicaid or MinnesotaCare	2406	Cost, low awareness, access, psychosocial	Free access, comprehensive treatment, e.g., NRT, behavioral counseling	OR = 1.47 (95% CI: 1.12–1.93)	High
Galiatsatos et al. [42]	Cohort	Tobacco clinics in public housing+ medication	Disadvantaged Public housing resident smokers	47	Stress, socioeconomic status	Community-based clinic support	94.4%	Low
Lee, J. et al. [43]	Cohort	Cessation advice receipt	U.S. adult smokers	6742	Lack of provider engagement	Not identified	OR = 0.99 (95% CI: 0.95–1.00)	Low
Kerkvliet et al. [44]	Cohort	Quitline service + cessation treatment	Smokers with and without mental health issues	4935	Psychological barriers	Not identified	OR = 0.78 (95% CI: 0.68–0.89)	Moderate
Wewers et al. [45]	Cohort	Cessation treatment involving community health worker + NRT	Appalachian resident smokers	707	Low health resource rural area	Social support	OR = 1.04 (95% CI: 1.01, 1.06)	Low
**(B)**								
**Author**	**Study Design**	**Intervention**	**Study Population**	**Sample Size**	**Identified Barriers**	**Identified Facilitators**	**Cessation** **Outcomes Effect, 95% CI**	**Quality Rating**
Kurti et al. [11]	RCT	Smartphone-based incentives	Pregnant smokers	30	Not identified	Smartphone-based incentivized intervention	OR = 9.33 (95% CI: 1.87–46.68)	High
Bailey et al. [12]	Cohort	ACA Medicaid expansion cessation	Medicaid-insured smokers	Expansion = 27,670 Non-expansion = 27,670	Limited access to healthcare due to insurance coverage	Medicaid expansion policies	OR = 1.35 (95% CI: 1.28–1.43)	High
Chen Lyu et al. [9]	Cohort	Social media-based cessation	Young adult smokers	248	Digital access barriers	Online peer support	OR = 2.6 (95% CI: 1.8–4.2)	High
Hooper, M.W. et al. [46]	RCT	Cessation treatment CBT/GHE	Treatment-seekers facing racial barriers	347	Racial discrimination	Coping response training	OR = 0.97 (95% CI: 0.94–0.99)	High
McCarthy et al. [47]	Cohort	EHR-enabled smoking treatment program	Primary care patients	6672	Access barriers	Automated prompts	OR = NP (95% CI: 2.2–10.5)	Moderate
Kreuter et al. [14]	RCT	Specialized quitline services with navigation	Low-income smokers	1137	Low awareness of NRT, lack of social support, financial	Specialized quitline compared to standard	OR = 0.70 (95% CI = 0.50–0.95)	High
Thrul [13]	RCT	Facebook-based live counseling	Young adult smokers aged 18–25 yrs	251	Low motivation and social support	Live counseling engagement	OR = 1.10 (95% CI 1.02–1.20)	High
Santiago-Torres et al. [48]	RCT	iCanQuit for American Indians and Alaska Natives	Indigenous smokers populations	169	Cultural barriers	Increase in acceptance of physical sensations, emotions, and thoughts	OR = 1.97 (95% CI: 0.92–4.25)	High
Santiago-Torres et al. [49]	RCT	ACT-based smartphone cessation	Rural smokers populations	550	Geographic isolation	Digital intervention	OR = 1.47 (95% CI: 0.96–2.27)	High
Webb Hooper et al. [50]	Observational	Cognitive-behavioral therapy (CBT) + 8 weeks nicotine patch (TNP)	Treatment-seeking smokers	234	Higher distress (perceived stress and depressive symptoms)	Reduction in distress via behavioral skills	OR = 0.93 (95% CI: 0.89–0.98)	Moderate

## Data Availability

Not applicable.

## Appendix A

**Table A1:** Search strategy.

Search Details	Results	Database
((“Tobacco Use Cessation”[MeSH Terms] OR “Smoking Cessation”[MeSH Terms] OR “Smoking Reduction”[MeSH Terms] OR (“Tobacco Control”[MeSH Terms] OR “Smoking Reduction”[MeSH Terms]) OR “Nicotine Replacement Therapy”[MeSH Terms]) AND (“outcome assessment, health care”[MeSH Terms] OR “quality assurance, health care”[MeSH Terms] OR “Treatment Outcome”[MeSH Terms] OR “Program Evaluation”[MeSH Terms] OR “Health Services Accessibility”[MeSH Terms]) AND (“Healthcare Disparities”[MeSH Terms] OR “Health Status Disparities”[MeSH Terms] OR “Health Inequities”[MeSH Terms] OR “Socioeconomic Disparities in Health”[MeSH Terms] OR (“Social Determinants of Health”[MeSH Terms] OR “Health Information Management”[MeSH Terms] OR “Health Impact Assessment”[MeSH Terms]) OR “Health Equity”[MeSH Terms] OR “Vulnerable Populations”[MeSH Terms] OR (“Minority Health”[MeSH Terms] OR “Race Factors”[MeSH Terms]))) AND (y_10[Filter])	50	PubMed
((‘tobacco use cessation’/exp OR ‘tobacco use cessation’ OR ‘smoking cessation’/exp OR ‘smoking cessation’ OR ‘tobacco control’/exp OR ‘tobacco control’ OR ‘smoking reduction’/exp OR ‘smoking reduction’ OR ‘nicotine replacement therapy’/exp OR ‘nicotine replacement therapy’) AND [2015,2016,2017,2018,2019,2020,2021,2022,2023,2024,2025]/py) AND ((‘outcome assessment, health care’/exp OR ‘outcome assessment, health care’ OR ‘quality assurance, health care’/exp OR ‘quality assurance, health care’ OR ‘treatment outcome’/exp OR ‘treatment outcome’ OR ‘program evaluation’/exp OR ‘program evaluation’ OR ‘health services accessibility’/exp OR ‘health services accessibility’) AND [2015,2016,2017,2018,2019,2020,2021,2022,2023,2024,2025]/py) AND ((‘healthcare disparities’/exp OR ‘healthcare disparities’ OR ‘health status disparities’/exp OR ‘health status disparities’ OR ‘health inequities’/exp OR ‘health inequities’ OR ‘socioeconomic disparities in health’/exp OR ‘socioeconomic disparities in health’ OR ‘social determinants of health’/exp OR ‘social determinants of health’ OR ‘health information management’/exp OR ‘health information management’ OR ‘health impact assessment’/exp OR ‘health impact assessment’ OR ‘health equity’/exp OR ‘health equity’ OR ‘vulnerable populations’/exp OR ‘vulnerable populations’ OR ‘minority health’/exp OR ‘minority health’ OR ‘race factors’/exp OR ‘race factors’) AND [2015,2016,2017,2018,2019,2020,2021,2022,2023,2024,2025]/py)	936	Embase
((((TS = (smoking cessation)) OR TS = (Smoking Reduction)) OR TS = (Tobacco Control)) OR TS = (Tobacco Use Cessation)) OR TS = (Nicotine Replacement Therapy) and Preprint Citation Index (Exclude–Database) AND ((((TS = (Health Care Outcome Assessment)) OR TS = (Health Care Quality Assurance)) OR TS = (Treatment Outcome)) OR TS = (Program Evaluation)) OR TS = (Health Services Accessibility) and Preprint Citation Index (Exclude–Database) AND ((((((((((TS = (Healthcare Disparities)) OR TS = (Health Status Disparities)) OR TS = (Health Inequities)) OR TS = (Socioeconomic Disparities in Health)) OR TS = (Social Determinants of Health)) OR TS = (Health Information Management)) OR TS = (Health Impact Assessment)) OR TS = (Health Equity)) OR TS = (Vulnerable Populations)) OR TS = (Minority Health)) OR TS = (Race Factors) Preprint Citation Index (Exclude–Database) and 2015 or 2016 or 2017 or 2018 or 2019 or 2020 or 2021 or 2022 or 2023 or 2024 or 2025 (Publication Years) and Article or Review Article (Document Types)	1426	Web of Science

**Table A2:** PRISMA Checklist.

Section and Topic	Item #	Checklist Item	Location Where Item Is Reported
TITLE			
Title	1	Identify the report as a systematic review.	Line 2–3, Page 1
**ABSTRACT**			
Abstract	2	See the PRISMA 2020 for Abstracts checklist.	The abstract section was prepared accordingly.
**INTRODUCTION**			
Rationale	3	Describe the rationale for the review in the context of existing knowledge.	Lines 63–91
Objectives	4	Provide an explicit statement of the objective(s) or question(s) the review addresses.	Lines 91–98
**METHODS**			
Eligibility criteria	5	Specify the inclusion and exclusion criteria for the review and how studies were grouped for the syntheses.	Lines 101–126
Information sources	6	Specify all databases, registers, websites, organisations, reference lists and other sources searched or consulted to identify studies. Specify the date when each source was last searched or consulted.	Lines 109–115
Search strategy	7	Present the full search strategies for all databases, registers and websites, including any filters and limits used.	Lines 109–115 and Appendix A Table A1
Selection process	8	Specify the methods used to decide whether a study met the inclusion criteria of the review, including how many reviewers screened each record and each report retrieved, whether they worked independently, and if applicable, details of automation tools used in the process.	Lines 109–126 and Figure 1
Data collection process	9	Specify the methods used to collect data from reports, including how many reviewers collected data from each report, whether they worked independently, any processes for obtaining or confirming data from study investigators, and if applicable, details of automation tools used in the process.	Lines 109–126 and Figure 1
Data items	10a	List and define all outcomes for which data were sought. Specify whether all results that were compatible with each outcome domain in each study were sought (e.g., for all measures, time points, analyses), and if not, the methods used to decide which results to collect.	Table 1A and Table 2B
	10b	List and define all other variables for which data were sought (e.g., participant and intervention characteristics, funding sources). Describe any assumptions made about any missing or unclear information.	Table 1A and Table 2B
Study risk of bias assessment	11	Specify the methods used to assess risk of bias in the included studies, including details of the tool(s) used, how many reviewers assessed each study and whether they worked independently, and if applicable, details of automation tools used in the process.	Lines 127–130
Effect measures	12	Specify for each outcome the effect measure(s) (e.g., risk ratio, mean difference) used in the synthesis or presentation of results.	Table 1A and Table 2B
Synthesis methods	13a	Describe the processes used to decide which studies were eligible for each synthesis (e.g., tabulating the study intervention characteristics and comparing against the planned groups for each synthesis (item #5)).	Lines 117–126 and Figure 1
	13b	Describe any methods required to prepare the data for presentation or synthesis, such as handling of missing summary statistics, or data conversions.	NA
	13c	Describe any methods used to tabulate or visually display results of individual studies and syntheses.	Table 1A and Table 2B
	13d	Describe any methods used to synthesize results and provide a rationale for the choice(s). If meta-analysis was performed, describe the model(s), method(s) to identify the presence and extent of statistical heterogeneity, and software package(s) used.	Lines 133–136
	13e	Describe any methods used to explore possible causes of heterogeneity among study results (e.g., subgroup analysis, meta-regression).	NA
	13f	Describe any sensitivity analyses conducted to assess robustness of the synthesized results.	NA
Reporting bias assessment	14	Describe any methods used to assess risk of bias due to missing results in a synthesis (arising from reporting biases).	Lines 133–136
Certainty assessment	15	Describe any methods used to assess certainty (or confidence) in the body of evidence for an outcome.	NA
**RESULTS**			
Study selection	16a	Describe the results of the search and selection process, from the number of records identified in the search to the number of studies included in the review, ideally using a flow diagram.	Figure 1
	16b	Cite studies that might appear to meet the inclusion criteria, but which were excluded, and explain why they were excluded.	Lines 143–147
Study characteristics	17	Cite each included study and present its characteristics.	Table 1A and Table 2B
Risk of bias in studies	18	Present assessments of risk of bias for each included study.	Table 1A and Table 2B
Results of individual studies	19	For all outcomes, present, for each study: (a) summary statistics for each group (where appropriate) and (b) an effect estimate and its precision (e.g., confidence/credible interval), ideally using structured tables or plots.	Table 1A and Table 2B; Figure 1
Results of syntheses	20a	For each synthesis, briefly summarise the characteristics and risk of bias among contributing studies.	Table 1A and Table 2B
	20b	Present results of all statistical syntheses conducted. If meta-analysis was done, present for each the summary estimate and its precision (e.g., confidence/credible interval) and measures of statistical heterogeneity. If comparing groups, describe the direction of the effect.	NA
	20c	Present results of all investigations of possible causes of heterogeneity among study results.	Lines 320–344
	20d	Present results of all sensitivity analyses conducted to assess the robustness of the synthesized results.	NA
Reporting biases	21	Present assessments of risk of bias due to missing results (arising from reporting biases) for each synthesis assessed.	NA
Certainty of evidence	22	Present assessments of certainty (or confidence) in the body of evidence for each outcome assessed.	NA
**DISCUSSION**			
Discussion	23a	Provide a general interpretation of the results in the context of other evidence.	Lines 377–390
	23b	Discuss any limitations of the evidence included in the review.	Lines 391–399
	23c	Discuss any limitations of the review processes used.	Lines 391–399
	23d	Discuss implications of the results for practice, policy, and future research.	Lines 401–411
**OTHER INFORMATION**			
Registration and protocol	24a	Provide registration information for the review, including register name and registration number, or state that the review was not registered.	Not registered
	24b	Indicate where the review protocol can be accessed, or state that a protocol was not prepared.	Not prepared
	24c	Describe and explain any amendments to information provided at registration or in the protocol.	Not registered/not prepared
Support	25	Describe sources of financial or non-financial support for the review, and the role of the funders or sponsors in the review.	NA
Competing interests	26	Declare any competing interests of review authors.	NA
Availability of data, code and other materials	27	Report which of the following are publicly available and where they can be found: template data collection forms; data extracted from included studies; data used for all analyses; analytic code; any other materials used in the review.	NA
